# Sexual Function in Pelvic Floor Disorders: A Pilot Study on the Feasibility of Routine Assessment

**DOI:** 10.3390/jcm15062131

**Published:** 2026-03-11

**Authors:** Esther Patricia Escamilla Galindo, Alicia Inmaculada Martín Martínez

**Affiliations:** 1 Department of Gynaecology and Obstetrics, Hospital Complex Materno-Infantil of Las Palmas de Gran Canaria, Av. Marítima s/n, 35016 Las Palmas de Gran Canaria, Spain; 2 Department of Medical and Surgical Sciences, Faculty of Health Sciences, University of Las Palmas de Gran Canaria, Juan de Quesada, 30, 35001 Las Palmas de Gran Canaria, Spain

**Keywords:** pelvic floor disorders, pelvic organ prolapse, urinary incontinence, sexual dysfunction, desire, quality of life, anxiety

## Abstract

**Background/Objectives**: Pelvic floor disorders (PFDs), which include pelvic organ prolapse and urinary incontinence, are common conditions that often affect sexual health, but remain under-assessed within routine care. The following cases are presented to demonstrate the potential of a brief sexual health questionnaire in pelvic floor clinics and to explore how sexual function varies across common PFD phenotypes. **Methods**: A pilot case series was conducted with a group of five sexually active women diagnosed with PFDs at the Materno-Infantil University Hospital in Gran Canaria, Spain, between January and December 2025. Patients completed the Female Sexual Function Index (FSFI) at the index visit. **Results**: Mean age was 40.6 years (range 35–46), mean parity was 1.6 births and 60% were active smokers. Mean FSFI total score was 26.9 (range 21.4–32.2) and 60% scored below 26.55. Desire and arousal were relatively preserved (means 5.0 and 4.9), whereas lubrication (3.4) and satisfaction (3.9) showed greater variability. Pain scores were low overall (mean 5.2). Self-rated sexual satisfaction was low in 40%, moderate in 40% and high in 20%. Moderate-to-high anticipatory sexual anxiety was present in 80%. **Conclusions**: Integrating a concise questionnaire based on the FSFI into the pelvic floor consultation appears to be a reasonable approach, with the potential to address secondary sexual dysfunction in patients with PFD, thereby facilitating personalised counselling and treatment.

## 1. Introduction

Pelvic floor disorders (PFDs), notably pelvic organ prolapse (POP) and urinary incontinence (UI), are highly prevalent conditions [1,2,3] encountered across women’s health services. However, it is noteworthy that only a small proportion of urogynecologists systematically assess women for sexual health concerns [4,5,6,7]. The classification of UI types is based on the criteria established by the International Continence Society, which divides them into three groups: stress UI, urge UI and mixed UI [8]. Impaired quality of life was found to be associated with all types of UI [1,2,8,9,10,11] and with diminished sexual desire, arousal, and satisfaction [12,13]. The anatomical compartment involved (bladder; uterus or the vaginal cuff after a hysterectomy; bowel) and the level of the prolapse are the two factors used to diagnose POP in the subtypes: anterior, apical, and posterior [14].

According to the World Health Organization, sexual health encompasses physical, psychological and social well-being aspects linked to sexuality. The term ‘sexual response’ was first coined in 1966 by Masters and Johnson in a pioneering study that laid the foundation for the field of sexual science [15,16]. The term refers to the set of psychophysiological changes that occur during sexual intercourse [17].

Sexual function in this context is inherently multidimensional, spanning desire, arousal, lubrication, pain, orgasm, satisfaction, and cognitive-emotional responses such as worry or avoidance [17,18]. Yet, in busy gynecologic clinics, the assessment of these domains is often fragmented or omitted [1], and the focus of conversation is frequently oriented towards the manifestation of pelvic symptoms, rather than encompassing a more comprehensive perspective on sexual health [1,5,6,7,18]. This disconnect is clinically important: sexual function constitutes a central component of quality of life and relationships, and it can influence help-seeking, adherence, and perceived benefit from treatment [7,19].

As has been previously reported, women suffering from POP tend to hold a negative perception of their vagina, which can engender feelings of insecurity regarding their partner’s sexual performance [3,11]. The most significant factors contributing to this negative impact on sexual domains include concerns about the presence of POP during intercourse, discomfort due to POP, and a diminished genital sensation [3,11]. In contrast, women experiencing UI often experience feelings of embarrassment surrounding their incontinence and pad use. Moreover, concerns regarding the odor of urine and the prospect of urine leakage during intercourse can hinder desire, arousal, and orgasm, potentially leading to dyspareunia.

The Female Sexual Function Index (FSFI) is a 19-item self-report questionnaire that is widely utilized as a measurement instrument to evaluate sexual function and its domains [4,20]. Lower scores in certain domains and the total FSFI score are indicative of worse sexual function. The instrument has received a “grade A” recommendation from the International Continence Society [4,21]. Despite the existence of valid and validated instruments [21], their utilization can be perceived as burdensome, and clinicians may experience uncertainty regarding the interpretation of domain scores for shared decision-making [5,6,7]. As a result, sexual well-being is frequently ignored in urogynecologic decision pathways. Nevertheless, it is a directly modifiable aspect that can be addressed through, patient-centered counselling, conservative measures (e.g., lubrication strategies, pain management), and the alignment of treatment expectations [3,7,11,16].

In this context, the implementation of a structured questionnaire, such as the FSFI or even specific questionnaires for this condition, such as the Pelvic Organ Prolapse/Sexual Incontinence Questionnaire (PISQ) [22], administered at the point of care has the potential to make sexual health visible within the context of the pelvic floor history [4]. Furthermore, it can be used to map which domains are most affected for a given phenotype [3,8,11,18].

The primary objective of this study was to demonstrate the feasibility and clinical utility of a validated sexual health questionnaire in a real-world pelvic floor clinic. To this end, a descriptive case series was conducted as a pilot feasibility study to explore the use of the FSFI in routine gynecological consultations for women with PFDs. A secondary objective was to demonstrate how diverse PFDs phenotypes can manifest alterations in sexual functions.

## 2. Materials and Methods

### 2.1. Study Design and Participants

A descriptive pilot case series was conducted involving five women presenting with symptomatic PFDs, between January and December 2025, at a public gynecology unit of the Materno-Infantil University Hospital in Gran Canaria, Spain. The study was designed to assess the feasibility of integrating a systematic sexual function assessment into routine clinical practice. Consecutive women with symptomatic PFDs attending routine visits were screened. Inclusion criteria were: (i) women aged >18 years; (ii) clinically diagnosed with symptomatic PFDs, including UI and/or POP; and (iii) currently sexually active. The exclusion criteria included inability to understand the questionnaire or refusal to participate. Women with conditions or prior treatments (such as psychiatric or neuromuscular disorders, previous obstetric or gynaecological surgery, pelvic radiotherapy or chemotherapy, or malignant disease) were excluded at the point of recruitment. It is well recognised that these factors substantially affect female sexual function, and they were beyond the scope of this pilot feasibility study [4,15,16,23,24].

### 2.2. Clinical and Sexual Function Assessment

Women were interviewed in a private consultation room at the gynecology outpatient clinic. Clinical and sociodemographic data, including age, parity, smoking status, educational level, and civil status, were abstracted from the electronic medical records. Following the initial anamnesis, urinary incontinence was evaluated through focused history-taking, pelvic examination, cough stress testing when needed, and assessment of pelvic floor muscle function, in accordance with standard clinical recommendations [25]. A conservative approach was adopted for all three UI cases, involving behavioural therapy, bladder training, pelvic floor muscle training and lifestyle modifications. In addition, a prescription for duloxetine was issued for mixed urinary incontinence.

POP was objectively evaluated using the Pelvic Organ Prolapse Quantification System (POP-Q) [11] during a standardized gynecological examination. The therapeutic approach employed in both cases involved the insertion of pessaries, with the dimensions of these devices being 59 mm and 65 mm, respectively.

Assessments were conducted at the index (initial) visit to our public gynecology service. Electronic records confirmed that no prior consultations for pelvic floor symptoms or documented sexual dysfunction had been recorded in the included cases.

Sexual function was assessed using the FSFI [4,20,21]. In addition to the standard FSFI, a specific item regarding anticipatory anxiety was administered to evaluate the level of distress specifically related to the prospect of sexual activity (“Do you feel anxious or worried when thinking about engaging in sexual activity?”). It has been demonstrated that symptoms associated with the pelvic floor have the potential to induce anticipatory anxiety and sexual avoidance [3,9,10,18,19].

### 2.3. Ethical Considerations

The study was conducted in accordance with the Declaration of Helsinki. The protocol was approved by the University Ethics Committee (CEIm HUGCDN: 2025-599-1). CEI/CEIMHospital Universitario Gran Canaria Dr. Negrín. During the initial consultation, the study objectives were clearly elucidated. All participants were informed that participation was voluntary and that their refusal would not affect their therapeutic management. Written informed consent was obtained from all subjects prior to data collection.

### 2.4. Statistical Analysis

Given the descriptive nature of this pilot feasibility study, data are presented as means, ranges, and raw scores. Cronbach’s α has been calculated, but it should be noted that this assumes tau-equivalence and unidimensionality. In the case of multidimensional scales, Cronbach’s α can over- or underestimate reliability and does not confirm a single-factor structure. Consequently, we also report domain-level McDonald’s ω, which offers a more appropriate reliability estimate for congeneric items [26,27]. No inferential statistical hypothesis testing was performed due to the limited sample size. All data were anonymized prior to database construction and analysis.

## 3. Results

### 3.1. Sociodemographic and Clinical Characteristics

Five women were included, with a mean age of 40.6 years (range 35–46 years) and a mean parity of 1.6 births. All women were premenopausal, and 60% (*n* = 3) were active smokers. Baseline characteristics are summarized in Table 1. With respect to the number of sexual partners, three women (60%) reported having had 10 or fewer, while the other two (40%) had had more than 10.

Regarding pelvic floor phenotypes, three women (60%) presented with UI, two had predominantly stress-related incontinence and one had mixed UI. The duration of symptoms was measured at 4, 5, and 12 months, respectively.

Of the two patients diagnosed with prolapse (40%), the first patient exhibited grade III anterior compartment POP, while the second patient demonstrated a combination of anterior compartment POP grades II and posterior compartment POP grade III. At the time of the initial visit, the duration of the symptoms was not documented.

The aforementioned women did not report any additional symptoms consistent with PFDs.

### 3.2. Sexual Function Profiles (FSFI)

The mean total FSFI score was 26.9 (range 21.4–32.2). Using the standard clinical cutoff of 26.55 for sexual dysfunction, 60% of the sample (*n* = 3) met the criteria for risk of sexual dysfunction. The findings of the FSFI investigation are presented in Table 2.

The internal consistency of the FSFI total score in our sample, calculated using the six domain scores (desire, arousal, lubrication, orgasm, satisfaction and pain), demonstrated acceptable reliability [28] (Cronbach’s α = 0.74), see Table 3.

Domain-level internal consistency (McDonald’s ω) is reported in Table 4.

Desire and arousal domains were relatively preserved across the cohort, with mean scores of 5.1 and 4.9 respectively.

Regarding lubrication and pain, these domains showed the greatest variability and clinical concern. For instance, one patient (mixed UI) reported a lubrication score of 4.5 and a pain score of 3.2, indicating certain degree of dryness and dyspareunia. Conversely, another patient (stress UI) reported excellent function with a total score of 32.2 and the mean maximal pain score in this domain was 6 (indicating no pain).

Self-rated satisfaction was low in 40% of the cases, moderate in 40%, and high in 20%, closely mirroring the total FSFI scores.

Notably, anticipatory anxiety regarding sexual activity was prevalent. 80% of women described always/often feeling anxious when thinking about engaging in sexual activity.

## 4. Discussion

There is a substantial number of publications that have been dedicated to demonstrating that female PFDs have a detrimental effect on sexual function [9,10,18,23,30,31]. Our pilot series confirms that sexual dysfunction is a common yet variable condition in women with PFDs, emphasizing the need for individualized care, and in order to investigate the impact of conservative, medical and surgical management of PFDs on sexual function, a comprehensive research effort is required [18].

A review study conducted by Verbeek and Hayward yielded findings indicating the prevalence of sexual dysfunction to be between 30% and 50% within the general female population. The prevalence of the condition was found to be elevated to a range between 50% and 83% among female subjects diagnosed with PFDs [9]. Zietarska-Cisak et al. reported that 64% of sexually active female patients consulting at urogynaecology clinics are affected by sexual dysfunction, driven largely by psychological factors rather than anatomical defects alone [4]. The present study is consistent with extant literature in identifying that 60% of the female participants, whether incontinence-related or associated with prolapse, have satisfied the criteria for sexual dysfunction (FSFI total score < 26.55) [8]. However, our findings reveal an important detail: a diagnosis of PFDs does not necessarily indicate sexual dysfunction, as evidenced by the findings of women in this study whose FSFI scores were 32.2 and 29.6, respectively. This underlines the importance of screening rather than assuming dysfunction.

A variety of well-established therapeutic interventions are available, from which a tailored treatment plan may be customised for the UI. A proposed scheme of treatment may include conservative measures, such as lifestyle changes, bladder training, Kegel exercises, physical therapy, biofeedback, electrostimulation, pharmacotherapy, pessaries, urethral bulking agents, mesh, and alternative surgical interventions. In the same line, non-surgical management for mild to moderate POP comprises prevention strategies, lifestyle advice, pessaries [25] and physiotherapy, with a particular emphasis on pelvic floor muscle training [14]. Nevertheless, even in these women undergoing conservative management, significant impairment of sexual function has been documented [23,32].

According to the findings of research conducted by Çancaya et al. on the subject, there is a demonstrable correlation between PFDs and impairment of sexual function, although the extent to which either POP or UI exerts the most deleterious effect remains equivocal [30]. This author recommends health professionals provide information on therapeutic alternatives to help women’s quality of life in the prevention and treatment of sexual dysfunction related to PFDs [30].

By contrast, within our cohort, the domains of desire (mean = 5.0), arousal (mean = 4.9), and pain (mean = 5.2) remained relatively preserved. However, the domains that were most affected were lubrication (mean = 3.4), orgasm (mean = 4.4), and satisfaction (mean = 3.9) (Table 2). These outcomes suggest that sexual impairment in women with PFD is not uniform and may depend on factors such as the severity of the dysfunction, the treatment modality (in this case, conservative) and the psychological and relational determinants of desire and arousal. This finding aligns with Tok et al., who concluded that the diagnosis of POP does not have a significant impact on specific sexual function domains, including sexual desire or satisfaction [23]. Conversely, as previously mentioned, the majority of literature on the subject indicates an overall reduction in sexual function domains in women diagnosed with PFDs [3,4,8,9,10,18,31]. Although desire is commonly cited as the domain most severely affected, Martins underscores substantial heterogeneity in which domains are impaired [11].

A key finding in our series was the high prevalence of moderate-to-high anticipatory sexual anxiety (80%), despite relatively preserved scores in the domains of desire and arousal. This suggests that the “drive” for intimacy remains, but it is hindered by anticipatory fear of symptoms (e.g., leakage during intercourse or dyspareunia). This pattern has been well described in women with PFDs [3,9,10,18,19].

Notwithstanding, the present study analyses a small sample of women characterized by mild to moderate symptoms and the absence of relevant comorbidities. This finding provides a potential explanation for the relatively preserved domains of desire and arousal. Moreover, sexual desire and arousal are influenced by psychological and relational factors that may persist despite the presence of PFDs [3]. This could explain the observed preservation in these domains within our sample. While lubrication and satisfaction scores were the most negatively affected.

The feasibility of our approach challenges the common barrier cited by clinicians, which is the lack of time [5,6,7,33]. As demonstrated in the pilot study, the utilization of a brief, coded panel based on the FSFI is highly recommended in a busy clinical setting. This validates our use of the domain-based scoring (FSFI) rather than a simple global score, as the latter may mask specific treatable issues like dyspareunia or dryness. This addresses a major gap in care highlighted by Neels et al., who have repeatedly shown that women across different life stages have poor knowledge of pelvic floor function and its sexual implications, and report limited information from healthcare providers [34,35].

### Limitations 

The primary limitation of this study is its small sample size (*n* = 5), which precludes statistical generalization. However, as a pilot feasibility study, the aim was not to infer causality but to validate the workflow. Cross-phenotype comparisons were not conducted.

The heterogeneity observed supports the need for larger, prospective cohorts to evaluate “stepped-care” interventions. Finally, the successful implementation of this pilot protocol paves the way for a prospective active intervention study.

Secondly, the cross-sectional design further limits the assessment of temporal changes in sexual function. Thirdly, the reliance on self-reported measures may be subject to recall bias. Fourthly, while PISQ-IR is condition-specific for UI/POP [22]; FSFI, a tool that has been validated and is widely utilized and useful for PFDs [8,13,23,30,32], was chosen to ensure cross-condition comparability for the purpose of future research, thereby facilitating a comprehensive comparison with urogynaecological sensitivity metrics. Nevertheless, the heterogeneity observed in this micro-cohort is indicative of the study’s pilot nature, thus providing valuable preliminary insights that underscore the necessity for larger, prospective cohorts to evaluate “stepped-care” interventions.

## 5. Conclusions

This pilot case series demonstrates that sexual dysfunction in women with PFDs is a heterogeneous condition that cannot be inferred solely from anatomical defects or pelvic symptoms. While sexual desire and arousal appeared relatively preserved in our cohort, suggesting a resilience of the sexual drive, we identified a critical burden of lubrication, sexual satisfaction and anticipatory anxiety deficits that significantly impair quality of life.

Our findings validate the hypothesis that the integration of a systematic, domain-based evaluation instrument, such as the FSFI, into routine urogynecological clinical practice is both feasible and clinically useful, effectively overcoming the perceived barrier of time constraints. By moving beyond a conventional questioning style, clinicians can uncover specific, treatable sexual dysfunctions that remain invisible in conventional anatomical assessments.

Based on these preliminary results, we advocate for a transition from passive screening to a “stepped-care” intervention model for PFDs clinics. The proposed model indicates that FSFI screening could facilitate the initiation of targeted, personalized therapeutic interventions for women experiencing sexual dysfunction. Future longitudinal research involving larger cohorts is required to evaluate the efficacy of this domain-informed counseling in improving patient-reported outcomes alongside surgical or conservative management of prolapse and incontinence.

## Figures and Tables

**Table 1:** Baseline characteristics of women.

Case	Phenotype	Age	BMI	Smoke	Parity	Education	Marital Status
1	Stress UI	35	32	Yes	1	Secondary	Separated
2	POP Cystocele grade III	43	29	Yes	2	Secondary	Stable relationship
3	Mixed UI	39	27	No	1	University	Stable relationship
4	Stress UI	40	30	No	1	University	Stable relationship
5	POP Cystocele grade II Rectocele grade III	46	33	Yes	2	Secondary	Stable relationship

Abbreviations: POP: Pelvic Organ Prolapse; UI: Urinary Incontinence; BMI: body mass index.

**Table 2:** Results of the FSF questionnaire.

Case	Desire		Arousal				Lubrication				Orgasm			Satisfaction			Pain			FSFI Total Score
	Q1	Q2	Q3	Q4	Q5	Q6	Q7	Q8	Q9	Q10	Q11	Q12	Q13	Q14	Q15	Q16	Q17	Q18	Q19	
1	5	4	4	4	4	5	4	4	3	4	2	2	2	1	1	1	3	2	3	21.4
2	4	4	4	4	4	4	1	2	1	1	2	2	5	5	4	5	5	5	5	26.3
3	5	5	5	5	4	5	4	4	3	4	4	5	5	4	3	4	5	5	5	32.2
4	4	2	5	3	3	3	2	2	2	2	5	5	5	3	3	3	4	5	4	25
5	5	4	4	4	3	5	3	4	3	4	3	4	3	5	4	5	5	5	5	29.6

Abbreviations: Q: question.

**Table 3:** Internal consistency reliability for dimensions and total FSFI (Cronbach’s α).

Sexual Domains	Cronbach’s Alpha	Interpretation ^1^
Desire	0.70	Acceptable
Arousal	0.57	Poor
Lubrication	0.98	Excellent
Orgasm	0.87	Good
Satisfaction	0.95	Excellent
Pain	0.95	Excellent
Total FSFI	0.74	Acceptable

^1^ Cronbach’s α values were interpreted according to the following established psychometric criteria: α ≥ 0.70: acceptable reliability; α ≥ 0.80: good reliability; α ≥ 0.90: excellent reliability [29].

**Table 4:** Internal consistency reliability for dimensions (McDonald’s ω).

Sexual Domains	McDonald’s Omega	Interpretation ^1^
Desire	0.71	Acceptable
Arousal	0.76	Acceptable
Lubrication	0.99	Excellent
Orgasm	0.89	Good
Satisfaction	0.96	Excellent
Pain	0.98	Excellent

^1^ Interpretation is based on commonly used psychometric thresholds (≥0.70 acceptable, ≥0.80 good, ≥0.90 excellent), applied to McDonald’s ω in line with previous methodological recommendations [26,27].

## Data Availability

The original contributions presented in this study are included in the article. Further inquiries can be directed to the corresponding author.

## Abbreviations

The following abbreviations are used in this manuscript:

PFDs/PFD	Pelvic Floor Disorders/Pelvic Floor Disorder
FSFI	Female Sexual Function Index
POP	Pelvic Organ Prolapse
UI	Urinary Incontinence
PISQ	Pelvic Organ Prolapse/Sexual Incontinence Questionnaire
POP-Q	Pelvic Organ Prolapse Quantification System
